# Biosensor characterization: formal methods from the perspective of proteome fractions

**DOI:** 10.1093/synbio/ysaf002

**Published:** 2025-02-12

**Authors:** Nicolás A Vaccari, Dahlin Zevallos-Aliaga, Tom Peeters, Daniel G Guerra

**Affiliations:** Laboratorio de Moléculas Individuales, Laboratorios de Investigación y Desarrollo, Facultad de Ciencias e Ingeniería, Universidad Peruana Cayetano Heredia, Lima 15102, Peru; Laboratorio de Moléculas Individuales, Laboratorios de Investigación y Desarrollo, Facultad de Ciencias e Ingeniería, Universidad Peruana Cayetano Heredia, Lima 15102, Peru; Open BioLab Brussels, Erasmushogeschool Brussel, Anderlecht, Brussels 1070, Belgium; Laboratorio de Moléculas Individuales, Laboratorios de Investigación y Desarrollo, Facultad de Ciencias e Ingeniería, Universidad Peruana Cayetano Heredia, Lima 15102, Peru

**Keywords:** biosensors, characterisation, dose-response, proteome, proteome fraction

## Abstract

Many studies characterize transcription factors and other regulatory elements to control gene expression in recombinant systems. However, most lack a formal approach to analyse the inherent and context-specific variations of these regulatory components. This study addresses this gap by establishing a formal framework from which convenient methods are inferred to characterize regulatory circuits. We modelled the bacterial cell as a collection of proteome fractions. Deriving the time-dependent proteome fraction, we obtained a general theorem that describes its change as a function of its expression fraction, a specific portion of the total biosynthesis flux of the cell. Formal deduction reveals that when the proteome fraction reaches a maximum, it becomes equivalent to its expression fraction. This equation enables the reliable measurement of the expression fraction through direct protein quantification. In addition, the experimental data demonstrate a linear correlation between protein production rate and specific growth rate over a significant time period. This suggests a constant expression fraction within this window. For an Isopropyl β- d-1-thiogalactopyranoside (IPTG) biosensor, in five cellular contexts, expression fractions determined by the maximum method and the slope method produced strikingly similar dose–response parameters when independently fit to a Hill function. Furthermore, by analysing two more biosensors, for mercury and cumate detection, we demonstrate that the slope method can be applied effectively to various systems. Therefore, the concepts presented here provide convenient methods for obtaining dose–response parameters, clearly defining the time interval of their validity and offering a framework for interpreting typical biosensor outputs in terms of bacterial physiology.

Graphical Abstract

**Figure:**
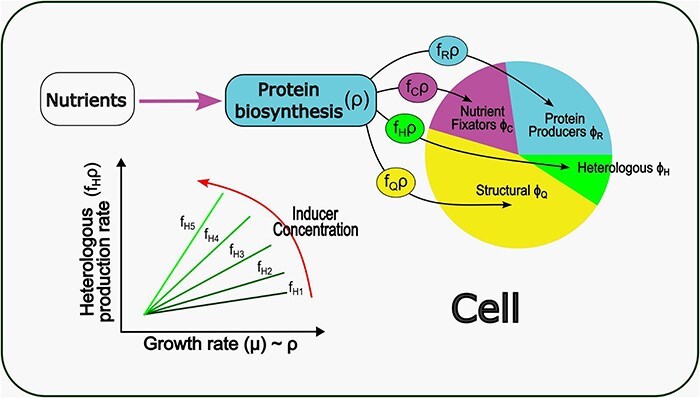
Nutrients, transformed by the action of the Nutrient Fixators (purple arrow), are used at a rate of ρ for Protein biosynthesis. The total rate ρ is multiplied by expression fractions f_R_, f_C_, f_H_, and f_Q_ to obtain the biosynthesis rate (black arrows) of each proteome fraction Φ_R_, Φ_C_, Φ_H_, Φ_Q_, respectively. In a graph of Growth rate versus Proteome Fraction Production Rate, a linear function (green lines) can be observed, and its slope is equal to the expression fraction at each condition.

Nutrients, transformed by the action of the Nutrient Fixators (purple arrow), are used at a rate of ρ for Protein biosynthesis. The total rate ρ is multiplied by expression fractions f_R_, f_C_, f_H_, and f_Q_ to obtain the biosynthesis rate (black arrows) of each proteome fraction Φ_R_, Φ_C_, Φ_H_, Φ_Q_, respectively. In a graph of Growth rate versus Proteome Fraction Production Rate, a linear function (green lines) can be observed, and its slope is equal to the expression fraction at each condition.

## 1 Introduction

Synthetic biology relies on the assumption that biological systems can be engineered by design, assembling parts and circuits like modules to create new systems of increasing complexity [1, 2]. However, regulatory circuits and even individual components often exhibit variable behaviours depending on the cellular context or culture conditions [3–7]. In addition, research groups employ different techniques and data analysis methods when characterizing these regulatory elements experimentally. This variation further hinders the ability to exchange parts in a modular approach.

Despite these challenges, the field has grown steadily. New biological parts are constantly being incorporated for novel engineered functions, leading to practical successes at different levels. For example, transcription factors and DNA or RNA sequences can link intracellular metabolites or extracellular signals to gene expression, controlling native or heterologous genes [8–10]. This strategy is applied to creating new biosynthetic pathways [11, 12], identifying optimal strains for the production of a molecule of interest [13, 14], and developing biosensors for various applications such as environmental monitoring, food safety, and disease diagnosis [15–18]. Furthermore, the activity of specific pathways can be regulated through transcription factors or sensing RNA elements, thus introducing a dynamic control to optimize metabolic flux towards the desired biosynthesis [19, 20].

There are a large number of characterized transcription factors, evaluated by their dose-response, detection limit, sensitivity, and dynamic range [20–23]. However, the lack of standardised procedures to analyse their ability to regulate reporter genes hinders progress in establishing robust libraries of modular tools. For example, there is no consensus on how to select culture time points that allow reliable measurement averaging between replicates or facilitate comparisons of output under different conditions, cellular contexts, and laboratories [24, 25].

This study addresses this challenge by proposing a theoretical framework for the characterization of regulatory circuits. This framework is based on the perspective that the cell is composed of a few proteome fractions, a concept that was used to study bacterial metabolism and growth regulation [7,26–33]. By mathematically defining the proteome fraction and deriving it over time, we link the evolution of a set of proteins to a specific fraction of the cell’s biosynthesis rate, which we call the expression fraction. From our basic assumptions, a simple theorem emerges, which suggests how to experimentally determine the value of the expression fraction using convenient and easily obtainable data, through two methods: directly by analysing a single point of peak accumulation of reporter protein or through the estimation of a linear relationship between specific production and specific growth rates during a given interval. We tested the reciprocal consistency of these two methods experimentally by comparing the dose–response profile of different *Escherichia coli* strains containing the DE3 production system when induced by varying IPTG concentrations and two other biosensors based on transcription factors, for the detection of mercury and cumate.

We consider that the framework presented in this article helps to tackle well-known challenges that are still prevalent in synthetic biology today [3, 34]. Firstly, it offers a theoretical basis for data analysis methodologies characterizing genetic circuits and their components, relying on easily measurable data such as culture growth and reporter protein levels. Secondly, our approach simplifies the mathematical modelling of genetic regulation systems by masking complexity through the concept of proteome fractions, while still capturing the interplay between native and heterologous cellular functions. This facilitates a deeper understanding of the inherent properties of sensing circuits, differentiating them from characteristics that depend on the cell and culture context. Finally, we speculate on how to extend this analysis to investigate orthogonality and potential crosstalk between heterologous and native genetic components.

## 2 Methods

### 2.1 *Escherichia coli* strains

MG1655 ΔendA ΔrecA (DE3) was a gift from Kristala Prather (Addgene code # 37854) [35]. BLR(DE3), and BL21(DE3) agar stabs were generously provided by Carlos Bustamante at UC Berkeley and Mauricio Baez at the Universidad de Chile, respectively. Cells were rendered competent by the $\mathrm{CaCl}_2$ incubation protocol and transformed by ${42}^\circ\mathrm{C}$ heat shock with pUC-T7GFP or simultaneously with pUC-T7GFP and pACYCDuet-1. Luria broth selection agar plates were supplemented with glucose 1% to reduce the basal expression of T7 and ampicillin to select cells transformed with the plasmid pUC-T7GFP or ampicillin and chloramphenicol to select those with the plasmids pUC-T7GFP and pACYCDuet-1.

### 2.2 Plasmids

Sequences were designed using Benchling RRID: SCR_013955 (Biology Software) (2020), retrieved from https://benchling.com. Twist Bioscience synthesized a gene block containing the following: T7 promoter, lac operator, Shine-Dalgarno ribosome binding sequence, a codon-optimised GFPmut3 coding sequence [36] fused with the LVA tag for rapid degradation (AANDENYALVA) [37], and the T7 terminator. The complete sequence is available in GenBank, accession number PQ015608.

A high copy number GFP expression plasmid, pUC-T7GFP, was built by inserting this construct into the pUC57 vector through standard digestion and ligation reactions between the restriction sites NcoI and KpnI. To introduce additional expression of LacI, cells were transformed with the low-copy plasmid pACYCDuet-1, acquired from GenScript.

The mercury biosensor was constructed by inserting the *merR* gene, which encodes the MerR mercury-sensitive transcription factor, and the promoter from transposon Tn501 from *Pseudomonas aeruginosa* plasmid pVS1 (GenBank: Z00027.1), containing the MerR DNA binding site, in the pUC57 vector. This enabled conditional expression of the *RFP* reporter gene in response to mercury. $\mathrm{NEB}^{\circledR}$ Stable *E. coli* competent cells were transformed with the resulting plasmid. The complete design, cloning procedures, and characterization are described in [38].

A cumate biosensor was constructed based on a regulatory circuit adapted from the study by Choi et al. [39]. Briefly, a constitutively expressed CymR protein represses a promoter that controls the expression of GFPmut3 fused with the LVA tag for rapid degradation (AANDENYALVA). Cumate-induced dissociation of CymR activates GFP expression. The circuit sequence was synthesized as a gBlock by Integrated DNA Technologies, Inc. and cloned at the NcoI and NotI sites of the pUC57 vector. The resulting plasmid was transformed into BL21(DE3), BLR(DE3), and MG1655(DE3) competent cells. The complete sequence is available in GenBank, accession number PQ010742.

### 2.3 Culture conditions

The specific growth rates and fluorescence data presented here were obtained from 200 *µ*l of microcultures in M9 medium supplemented with 0.2% casamino acids and 0.24% glucose, prepared as follows: freshly transformed colonies were handpicked to inoculate 10 ml of M9 medium supplemented with ampicillin, or ampicillin and chloramphenicol when pACYCDuet-1 was included. These cultures were incubated at $37^\circ\mathrm{C}$ and 250 RPM overnight, and their cell density was measured and harvested by centrifugation at 5000 RPM for 5 min. Immediately, cells were resuspended in fresh medium to the appropriate volume to obtain an optical density of 0.1 (approximately $2 \times 10^7$ cells$\cdot\mathrm{ml}^{-1}$) and were loaded in a 96-microtiter plate. At this point, reporter protein expression was induced in 195 *µ*l of culture by manually pipetting 5 *µ*l of the appropriate inducer ($\mathrm{IPTG, HgBr}_{2}$, cumate), reaching the final concentration indicated for each case, completing 200 *µ*l in each well. These microcultures were incubated at ${30}^{\circ}\mathrm{C}$ with oscillatory shaking at 220 RPM for 16 h without the addition of antibiotics. The microtiter plate was covered with its plastic lid and the outer wells were filled with 200 *µ*l of water so that a similar humidity surrounded all monitored cultures. This configuration resulted in a volume loss of less than 2.5% due to evaporation after 16 hours.

### 2.4 Measurements

The culture's optical density at 600 nm wavelength ($\mathrm{OD}_{600}$) and fluorescence were monitored in a plate reader (Tecan Infinite 200 Pro). The production of GFP was registered with the following settings: 475 nm excitation and 516 nm emission, with the signal gain set at 50 out of 100. Similarly, the production of RFP was recorded using 585-nm excitation and 608-nm emission, with the signal gain set at 95. In both cases, fluorescence data were collected with 25 flashes, reading every 15 min

### 2.5 Total protein quantification and visualisation

For this assay, two flasks (for each strain), containing 50 ml of transparent medium, were inoculated with the bacteria so that the final $\mathrm{OD}_{600}$ was 0.1. One of the flasks was induced with IPTG (0.5 mM) at the beginning of the culture. The cultures were then left to grow at $30^{\circ}\mathrm{C}$. Cultures were sampled at three time points: 0, 2, and 4 h. For each of the samples taken during bacterial growth, two tests were carried out: quantification of total proteins using the Bradford reagent and visualization of total proteins using a polyacrylamide gel. For the total protein quantification essay, 2 ml of culture was taken and the bacteria were pelleted at 13 500 rpm for 5 min. This pellet was then resuspended in 500 *µ*l of 8 M urea and lysed by three cycles of freeze-thawing for 15 min at $-70^{\circ}\mathrm{C}$ and 15 min at $37^{\circ}\mathrm{C}$. Finally, 10 *µ*l of this suspension was mixed with 300 *µ*l of Bradford reagent (Thermo Fisher Scientific), incubated 10 min at room temperature, and measured at 595 nm for protein quantification. To estimate the percentage of GFP relative to total proteins, we performed total protein electrophoresis and used the intensity of the bands to calculate it. To do so, 2 ml of culture was pelleted, resuspended in 500 *µ*l of 2% sodium dodecyl sulfate (SDS), and heated at $95^{\circ}\mathrm{C}$ for 10 min. Subsequently, this suspension was run on a 15% polyacrylamide gel.

### 2.6 Estimation of the heterologous fraction, specific growth rate, and specific production rate

We considered fluorescence directly proportional to the mass of the reporter protein (GFP or RFP) and the optical density proportional to the total protein mass. Thus, the ratio of fluorescence to optical density, $\frac{\text{flu}(t)}{{\text{OD}}_{600}(t)}$, was measured to estimate the heterologous fraction, *φ*_*H*_.

To calculate the specific growth rate at each time point $\mu(t)$, the optical density was measured at *t* and at the immediate next time point $t+\Delta t$. The difference between both measurements was divided by Δ*t* and the optical density at the former point *t*:


(1) $$ \mu \left(t\right)=\frac{{\text{OD}}_{600}(t+\Delta t)-{\text{OD}}_{600}(t)}{\Delta t}\cdot \frac{1}{{\text{OD}}_{600}(t)}. $$

The specific production rate of GFP (or RFP) was estimated by normalizing the slope of fluorescence accumulation over time. For GFPmut3, the fluorophore maturation time is 4.1 min [36], and therefore, no maturation delay was introduced in the calculations, as it is shorter than our measurement time window of 15 min. However, the merR system used RFP as a reporter which has a maturation time of 1 h, meaning that the red fluorescence observed in any given time is proportional to the amount of *RFP* produced 4 measuring points earlier. We corrected this by shifting the fluorescence curve back in time by 4 points. Finally, we calculated ${\rho }_\text{H}$ by measuring the fluorescence at time *t* and at next time point $t+\Delta t$. We divide the difference by time step Δ*t* and optical density at time *t*:


(2) $$ {\rho }_\text{H}\left(t\right)=\frac{{f\,lu}(t+\Delta t)-{f\,lu}(t)}{\Delta t}\cdot \frac{1}{{\text{OD}}_{600}(t)}. $$

### 2.7 Calculations

Data handling, processing, plotting, regression analysis, and modeling were performed using the R programming language. All linear and non-linear regressions were performed using the R built-in functions: lm and nls, respectively. The dataset employed was uploaded in figshare (DOI: https://doi.org/10.6084/m9.figshare.26314723.v1) [40].

To determine the expression fractions using the maximum method, the time series data for *φ*_*H*_ were grouped by strain and induction condition (*N* = 8 for BL21(DE3) with the DE3 system, *N* = 9 for the MerR and CymR systems, and *N* = 4 for all other cases). Subsequently, $\varphi^{\text{max}}_{\text{H}}$ was identified for the first 10 h of culture for each series within each group and these values were averaged. The average $\varphi^{\text{max}}_{\text{H}}$ was obtained at various inducer concentrations and then used in the Hill fitting process.

For the slope method, the expression fraction was determined as the slope obtained from a linear regression between the specific growth rate and the specific production rate during the growth phase (*µ* > 0). To establish the appropriate time window for this regression, all specific growth rate data for each strain under each induction condition were analysed. At each time point, a simple *t*-test was used to assess each time point to assess whether the average value of the specific growth rate differed significantly from zero (*N* = 8 for BL21(DE3) with the DE3 system, *N* = 9 for the MerR and CymR systems, and *N* = 4 for all other cases). If a significant difference was found (*α* = 0.05), the time point was considered part of the growth phase and included in the regression analysis. Subsequently, the earliest data points were iteratively excluded one by one until the determination coefficient (*R*^2^) reached a maximum value, which was > 0.9 in all cases. The slopes of the regressions obtained at various inducer concentrations were registered and used to fit the Hill function.

Dose–response profiles were used to build Hill functions for each biosensor. A scatterplot was created depicting the expression fractions data (obtained from slopes or $\varphi^{\text{max}}_{\text{H}}$ values) versus the inducer concentration for each titration experiment. The nls function with the port algorithm was then used to fit a Hill function to this plot. Lower bounds of 0 were set for the values of *H* (maximum expression) and $\ k_I$ (affinity constant), while a lower bound of 1 was set for the value of *n* (Hill coefficient). The weight of each data point was determined by the inverse of its variance, which assigned a higher weight to more precise measurements.

## 3 Results

### 3.1 DE3 system illustrates the challenges of biosensors characterization

We began our investigation experimenting with the DE3 expression system in B and K-12 *E. coli* strains. This system combines a native element, the LacI repressor, and a heterologous one, the T7 RNA polymerase (T7RNAP)[41], and is widely applied in the production of recombinant proteins [42]. In addition, the LacI repressor served as a model for biosensor studies [43], and both the LacI repressor and T7RNAP have been used as modular components in the construction of logic gates within synthetic regulatory circuits [44–46].

**Figure 1.**
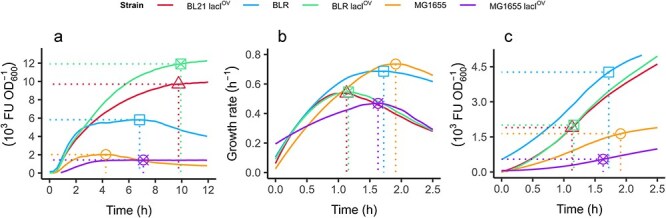
Selecting time points for analysing the heterologous proteome fraction (*φ*_*H*_). The images compare different criteria for selecting time points to analyse the heterologous proteome fraction in five different *E. coli* strains containing the DE3 system, $BL21\ {lacI}^{OV}$, *BLR*, $BLR\ {lacI}^{OV}$, $MG1655$, and $MG1655\ {lacI}^{OV}$. Cells co-transformed with pACYCDuet for the additional expression of LacI are indicated as $\mathrm{lacI}^{OV}$. (a) Specific fluorescence accumulation over 12 h of cultivation after induction. The markers indicate the point of maximum specific fluorescence, ${\varphi }^{\text{max}}_\text{H}$. (b) Specific growth vs. time; the markers indicate the maximum specific growth rate for each culture, and the dotted line is a visual aid to identify the time at which it occurs. (c) Specific fluorescence accumulation for the first 2.5 h of cultivation after induction by 1 mM IPTG. The markers indicate the maximum specific growth rate for each culture, and the dotted lines indicate the time and the value of the specific fluorescence at these points.

For our DE3 system experiments, we used a plasmid containing the GFP gene under the control of the T7 promoter and a LacI operator. Using this setting as an IPTG biosensor, we employed a two-stage culture protocol. The first stage was focused on the accumulation of biomass, and then in a subsequent phase, starting at $\mathrm{OD}_{600}$ = 0.1, the expression was induced by IPTG addition. This configuration, commonly used to optimize protein production or biosensor signal output, revealed variations in specific growth rates and GFP output both between different *E. coli* strains. Furthermore, because the sensitivity of transcriptional biosensors is influenced by the concentration of the transcription factor involved in the circuit, we introduced additional expression of the LacI repressor by cotransfecting each strain with pACYCDuet, resulting in further variations. Although differences between cultures are visible (Figure 1), analysing them requires applying objective criteria that are not self-evident.

Many researchers choose to measure fluorescence end points 24 h or more after induction, which corresponds to the stationary phase [47–49]. These easily identifiable time points can yield high signal output due to prolonged protein accumulation. However, we caution that late measurements may produce misleading quantitative comparisons among strains. In the results presented here, for MG1655(DE3) and BLR(DE3), the normalized fluorescence decreases after 4 and 6 h, respectively (Figure 1a), probably due to factors not related to the sensing circuit and protein biosynthesis. This can lead to potentially inaccurate interpretations of the circuit properties. Alternatively, through trial and error, the analysis can be shifted to an earlier time point, as is common in the case of recombinant protein production, where short induction times minimize the potentially undesirable consequences of heterologous overexpression [50–52], such as toxicity and protein misfolding.

In the field of metabolic engineering, measurements are typically taken during the exponential growth phase of microbial cultures, as this phase is generally associated with a stable metabolic state, improving assay reproducibility. Furthermore, if the goal is to identify highly productive circuits or strains, or to implement dynamic feedback control for a new, engineered pathway, it is preferable to characterize a period near the peak of the specific growth rate, as this is when the anabolic capacity is at its maximum. Specifically, measuring at the time of maximum growth rate is a widely accepted practice and provides a reliable and consistent point for data collection [33, 53, 54]. However, when observing different *E. coli*strains, it is clear that the peak of the specific growth rate does not align across cultures at any particular time point (Figure 1a), which means that different strains would have different times to accumulate the heterologous protein. In our case, even though all cultures started at the same cellular density and were stimulated with IPTG at the same time, BLR(DE3) and MG1655(DE3) showed their maximum specific growth rates later than BL21(DE3). Consequently, if these points are selected, the production of GFP by BL21(DE3) and BLR(DE3) with additional LacI would be plotted just after 30 min of induction, when the fluorescence is still relatively low, while those of BLR(DE3) and MG1655(DE3) would be evaluated after longer accumulation (Figure 1c). Although the peak of the specific growth rate is a common reference for metabolism and regulation studies, in our case, analysing the protein yield at these time points would lead to unfair comparisons. For example, strain MG1655(DE3) would appear to be a producer similar to B strains simply because the latter are analysed after a shorter protein accumulation time (Figure 1).

This case illustrates that analysing a culture’s response to heterologous expression requires identifying a time point or interval that offers biologically meaningful information, taking into account the induction strategy and the culture’s evolution over time. Given this circumstance, a new criterion is needed to select measurement points to reliably study genetic circuits, such as biosensors, in a manner that enables accurate comparisons and reproducibility, even across different strains.

### 3.2 A theorem for the evolution of proteome fractions in time: the concept of expression fraction

**Figure 2.**
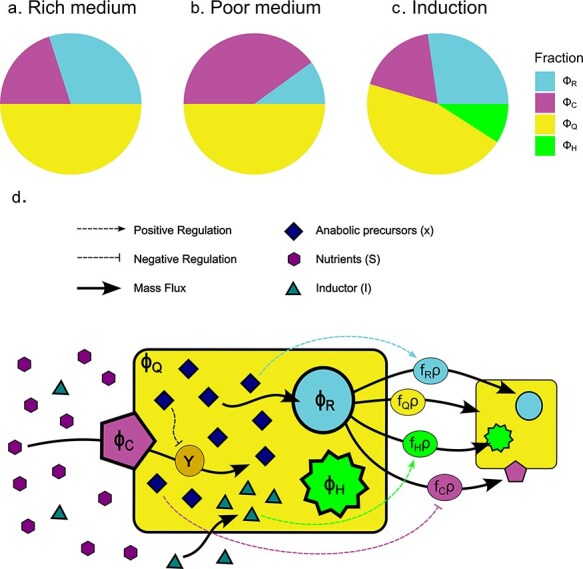
A visual representation of proteome fractions and the biosynthesis allocation model. Representation of the proteome fractioning of a cell: (a) in a rich medium, the cell maximizes its specific growth rate through the activity of the ribosomal fraction. In this context, the cell produces more of this type of protein, ${\varphi }_\text{R}$. (b) In a poor medium, the cell maximizes its fixation of nutrients through the activity of the carbon fixator fraction, *φ*_*C*_. Thus, via its own genetic regulation, the cell shifts its production towards *φ*_*C*_. (c) Producing a heterologous fraction, *φ*_*H*_, causes a redistribution of the native proteome allocation. (d) This image is a representation of nutrient fixation and protein biosynthesis rates within bacteria. Biochemical transformations fluxes are represented by solid arrows: the purple hexagons represent the nutrients *S*(*t*), which are converted to anabolic precursors, *x*(*t*), (dark blue diamonds) by the nutrient fixator, *φ*_*C*_, at a rate *Y*. The anabolic precursors are then consumed by the ribosomal fraction, ${\varphi }_\text{R}$, to produce new biomass (represented by the small cell on the right side) at a rate *ρ*. Total biosynthesis is divided into a multioutlet branching of specific protein production rates, each with their respective expression fractions, $f_\text{R}(t)$, $f_\text{C}(t)$, $f_\text{Q}(t)$, and $f_\text{H}(t)$. Dashed arrows and dashed blunt lines represent positive and negative regulations, respectively: The concentration of anabolic precursors stimulates the specific rate of ${\varphi }_\text{R}$ production and represses the specific rate of *φ*_*C*_ production. The specific production rate of ${\varphi }_\text{H}$ is activated by its specific inducer (green triangles), which diffuses from the extracellular space. The specific production rate of *φ*_*Q*_ is constant.

We argue that when analysing the production of heterologous proteins in general, and whole cell biosensors in particular, the focus should shift from protein yield to biosynthesis flux allocation. We conceptualized the general biosynthesis rate divided into a set of rates (Figure 2), each producing a different type of protein, termed a proteome fraction (*φ*_*i*_). Similarly as has been done in models previously reported [26–32], proteins will be grouped together as a unified proteome fraction if they share a similar biological function and are regulated by the same cues [26, 33]. In previous work, a proteome fraction that fulfils the core structural functions will remain constant (*φ*_*Q*_), while the other two fractions are variable and perform nutrient assimilation (${\varphi }_\text{C}(t)$) and biomass production (${\varphi }_R(t)$), respectively (Figures  2a and b). Finally, in the case of one set of heterologous proteins controlled by a single circuit, these will constitute a fraction called the heterologous fraction (${\varphi }_H(t)$ in Figure 2c). The total anabolic flux will be divided into the synthesis of specific protein sets, in proportions denoted as the expression fractions, represented by $f_i(t)$, where *i* is the protein group to which it is dedicated ($f_\text{R},\ f_\text{Q},\ f_\text{H},\ f_\text{C}$ in Figure 2d). In this way, the expression fraction *f_i_* can be understood as the proportion of total ribosomal activity that synthesizes the proteome fraction *φ*_*i*_. The value of $f_i(t)$ will depend on the specific regulatory mechanism of the protein set (represented as dashed lines in Figure 2d) and the external and internal conditions of the cell. The full conceptual and mathematical reasoning behind the expression fraction is detailed in the Supplementary data.

With these definitions, solving the derivation of a proteome fraction over time (complete derivation in the Biosynthesis Allocation Theorem section of the Supplementary data) produces an equation that links the change in a proteome fraction to its expression fraction and the total biosynthesis rate, $\rho (t)$, as follows:


(3) $$ \frac{\text{d}{\varphi }_i(t)}{\text{d}t}=\ \rho (t)\cdot [f_i(t)-{\varphi }_i(t)]. $$

In the subsequent sections, we will explain how the expression fraction of a heterologous protein $f_\text{H}(t)$ can be determined from easily obtainable experimental data.

### 3.3 Finding the expression fraction and its regulation: the maximum method

A remarkable consequence emerging from equation 3 is that for any proteome fraction, when its differential over time equals zero ($\frac{\text{d}{\varphi}_i(t)}{\text{d}t}=0$), the expression fraction and the proteome fraction must be equal $f_i(t)={\varphi }_i(t)$ (assuming that the cell’s anabolic rate is never zero as long as it is alive). Therefore, regardless of the growth behaviour of a culture, registering the heterologous fraction when it has reached a maximum should correctly represent the expression of the circuit. Although testing this prediction would require sophisticated techniques like isotopic labelling and quantitative proteomics, which is beyond our current scope, we can assess whether analysing biosensor output using the expression fraction provides meaningful information that enables biologically meaningful comparisons of circuit behaviour across strains. To do this, we collected data from the DE3 system (Figure 1) at the peak of normalized fluorescence accumulation. Data collected at this point under various conditions should be reliable for constructing a function representing regulated circuit expression, such as in response to varying inducer concentrations.

It is generally accepted that gene expression responds to the concentration of an inducer following a Hill function [55], a model commonly applied to biosensors [43]. Using a Hill equation for representing the regulation of the heterologous fraction as a function of the inducer *I*, we obtain the following equation (for more details, see Supplementary data):


(4) $$ {\varphi }^{\text{max}}_\text{H}(I)=f_\text{H}(I)=H\cdot \frac{I^n}{(H+1)\cdot I^n+{k_I}^n}. $$

Here, *H* represents the maximum possible expression for the circuit, *I* is the molecular inducer concentration, *k_I_* is related to the affinity for *I*, and *n* is the Hill coefficient that expresses the cooperativity of the response to *I*.

**Figure 3.**
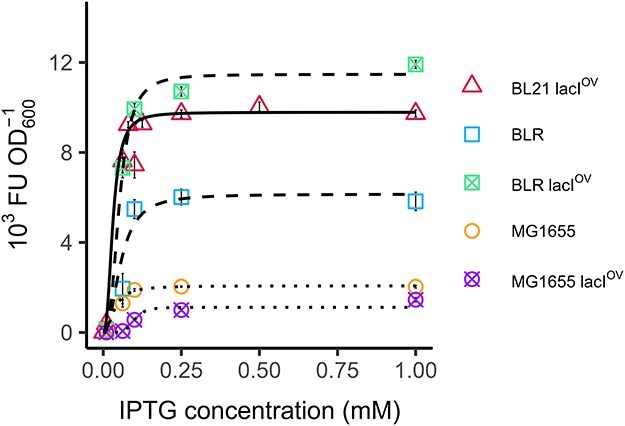
Dose–reponse diagrams for the DE3 system using the maximum method. The diagrams were made by plotting the maximum values of specific fluorescence obtained for six different IPTG concentrations across five different cellular contexts (*N* = 8 for BL21(DE3) and *N* = 4 for all other cases).

Now we will use the values of ${f_i}(t)$ obtained from the maximum values of the heterologous fraction, ${\varphi }^{\text{max}}_\text{H}$, as described earlier, to test if they follow a dose–response profile in the shape of a typical Hill function. First, we verified by SDS polyacrylamide gel electrophoresis analysis that even for the highest GFP overexpression, $\varphi_\text{H}$ represented < 15% of total biomass (Fig. 3S); therefore, we infer that in most cases, the coefficient $(H+1)$ can be approximated to 1, thus resulting in a typical Hill function. Importantly, because in this kind of titration experiment, the curves represent the behaviour of the whole cell, *k_I_* and *n* will not strictly correspond to the dissociation constant and the molecular cooperativity of the binding reaction between the transcription factor and the inducer.

For the DE3 expression system, the expression fractions, derived from experimental ${\varphi }^{\text{max}}_\text{H}$ values, related to the inducer concentration according to a Hill function (Figure 3). We achieved excellent fits in five different cell contexts, including three distinct *E. coli* strains with and without an additional LacI repressor. The parameters obtained from these fits are listed in Table 1. For comparison, data taken at the points of maximum growth rate and 12 h after induction (stationary phase) were used to plot dose–response diagrams and adjusted to a Hill function in Figure 1S; the corresponding parameters are shown in Table 1S.

**Table 1.** Hill function parameters describing the biosensors responses. The Hill function was fitted to the values of the expression fraction plotted as a function of inducer concentration (IPTG, cumate, or $\mathrm{HgBr}_{2}$). $f_\text{H}={\varphi }^{\text{max}}_\text{H}$$f_\text{H}=\frac{\Delta {\rho }_\text{H}}{\Delta \mu }$

		${\boldsymbol{H}} ({\text{flu}}/{{\text{OD}}}_{{600}})$		${\boldsymbol{k}}_{{I}} ({\mu } \text{M})$		*n*	
Sensor	Strain	Maxima^a^	Slopes	Maxima	Slopes	Maxima	Slopes
IPTG	${{BL21(DE3) + LacI}}^{OV}$	9784 $\mathrm{\pm}$ 191	8457 $\mathrm{\pm}$ 225	31.0 $\mathrm{\pm}$ 3.7	35.6 $\mathrm{\pm}$ 5.5	2.5 $\mathrm{\pm}$ 0.3	2.5 $\mathrm{\pm}$ 0.5
	BLR	6148 $\mathrm{\pm}$ 496	6668 $\mathrm{\pm}$ 651	55.8 $\mathrm{\pm}$ 12.9	58.0 $\mathrm{\pm}$ 14.9	2.2 $\mathrm{\pm}$ 0.3	2.7 $\mathrm{\pm}$ 0.5
	${{BLR + LacI}}^{OV}$	11 473 $\mathrm{\pm}$ 319	11 080 $\mathrm{\pm}$ 563	51.7 $\mathrm{\pm}$ 5.1	67.7 $\mathrm{\pm}$ 6.9	2.8 $\mathrm{\pm}$ 0.3	4.4 $\mathrm{\pm}$ 1.8
	MG1655(DE3)	2072 $\mathrm{\pm}$ 44	2846 $\mathrm{\pm}$ 438	36.6 $\mathrm{\pm}$ 4.1	56.3 $\mathrm{\pm}$ 22.8	2.1 $\mathrm{\pm}$ 0.2	2.0 $\mathrm{\pm}$ 0.7
	${{MG1655(DE3) + LacI}}^{OV}$	1116 $\mathrm{\pm}$ 116	1747 $\mathrm{\pm}$ 97	99.8 $\mathrm{\pm}$ 4.5	93.6 $\mathrm{\pm}$ 2.6	5.9 $\mathrm{\pm}$ 1.4	6.6 $\mathrm{\pm}$ 0.5
Cumate	BL21(DE3)	1170 $\mathrm{\pm}$ 84	2965 $\mathrm{\pm}$ 110	89.6 $\mathrm{\pm}$ 13.4	79.7 $\mathrm{\pm}$ 6.1	2.5 $\mathrm{\pm}$ 0.2	4.3 $\mathrm{\pm}$ 0.6
	BLR(DE3)	989 $\mathrm{\pm}$ 45	2390 $\mathrm{\pm}$ 33	50.8 $\mathrm{\pm}$ 5.3	33.5 $\mathrm{\pm}$ 1.8	2.3 $\mathrm{\pm}$ 0.2	4.0 $\mathrm{\pm}$ 0.2
	MG1655(DE3)	1539 $\mathrm{\pm}$ 69	2575 $\mathrm{\pm}$ 127	243 $\mathrm{\pm}$ 17	208 $\mathrm{\pm}$ 15.3	1.8 $\mathrm{\pm}$ 0.2	3.2 $\mathrm{\pm}$ 0.5
${{Hg}}^{2+}$	${{NEB}}^{\circledR}$ Stable	-	11 722 $\mathrm{\pm}$ 3130	-	554 $\mathrm{\pm}$ 281 (nM)	-	0.99 $\mathrm{\pm}$ 0.1

a The expression fractions were determined as equal to the heterologous protein fraction at its maximum value, $f_H=\varphi_H^{max}$

b The expression fractions were determined by measuring the slope within the linear interval of the graph of specific production rate against specific growth rate, $f_\text{H}=\frac{\Delta {\rho }_\text{H}}{\Delta \mu }$

### 3.4 Finding the expression fraction during growth: the slope method

In the cases analysed, we observed that the maximum point for the heterologous fraction is reached close to or during the stationary phase. Because there are physiological changes in the onset of this phase, there is no guarantee that the biosynthesis fraction dedicated to our gene of interest will remain constant in other stages of the culture. Moreover, as stated previously, it is important for biosensor developers, especially in the field of metabolic engineering, to have a solid method to characterize heterologous expression during growth.

We aim to develop an effective analysis strategy by linking the expression fraction to the specific growth rate. By definition, the biosynthesis rate of the heterologous protein fraction, $\rho_\text{H} (t)$, is the product of its expression fraction, *f*_H_ and the total protein biosynthesis rate, ${\rho }(t)$:


(5) $$ {\rho }_\text{H}(t)=f_\text{H}(t)\cdot \rho (t). $$

During exponential growth, the ratio between protein and total cell mass remains relatively constant [56]. Therefore, we can confidently assume that the total protein biosynthesis rate remains linear to the specific growth rate, which implies that we can substitute $\rho (t)$ with $\mu(t)$ without altering the proportionality of change. Since measuring the specific growth rate directly is simpler than determining the total biosynthesis rate of the cell, this approximation yields a convenient equation:


(6) $$ \ \rho_\text{H}(t)=\ f_\text{H}(t) \cdot \mu (t). $$

**Figure 4.**
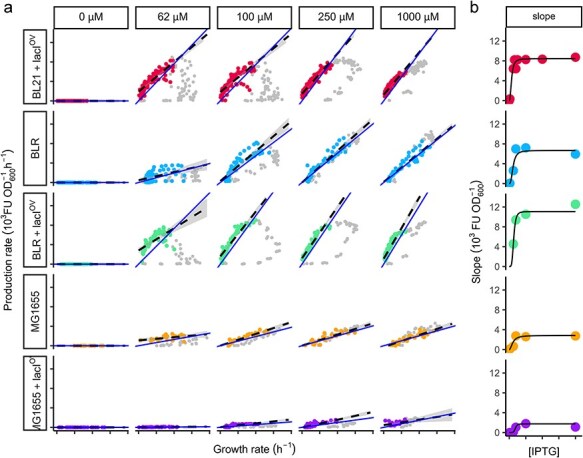
Specific production rate vs. specific growth rate plots and dose–response diagrams using the slope method. (A) Experimental data for each inducer concentration and *E. coli* strain were plotted separately. A time window (coloured dots) was selected to exclude earlier points (grey dots) and fit a linear regression (dashed line). The time window maximized the coefficient of determination ($\mathrm{R}^{2}$), as described in the Methods section. Blue solid lines represent linear functions with slopes equal to ${\varphi }^{\text{max}}_\text{H}$, visualizing the close agreement between expression fraction values determined by the slope and maximum methods. (B) Dose–response diagrams were generated by plotting the slope values from the linear regressions against their corresponding IPTG concentrations. A Hill function (black line) was fit to each dose–response curve.

It follows that *f*_H_ can be experimentally determined as equal to the slope in a plot of the specific production rate of the heterologous protein vs specific growth rate of the culture. We graphed the fluorescence-specific production rate (${\rho }_\text{H}$) against the specific growth rate (*µ*) at all time points in Figure 4a. Following induction, our initial data points from each culture show a curved line (grey dots) resembling a hook. This curved line transitions into a straighter segment (coloured dots) that can be approximated by a linear relationship. We interpret this initial curvature as the time it takes for the inducer and the regulatory machinery to reach a steady state. Interestingly, the maximum growth rate falls within this nonequilibrated time. The following straighter segment, on the other hand, represents a period with a constant slope or expression fraction (*f*_H_) of the heterologous protein.

We used the resulting *f*_H_ values to generate dose–response diagrams for each strain. Similar to the analysis performed with the values obtained from the ${\varphi }^{\text{max}}_\text{H}$ method, each dose–response diagram was used to fit a Hill function Figure 4b. The parameters obtained from these fits are also listed in Table 1.

Another important step is to investigate whether the *f*_H_ values obtained from ${\varphi}^{\text{max}}_\text{H}$ are similar to those obtained from the slopes. This would indicate that *f*_H_ remains constant throughout the interval in which the linear trend is maintained, including a significant portion of exponential growth and the beginning of the stationary phase. In Figure 4a we present a visual comparison of *f*_H_ values determined from the slope of the specific production versus the specific growth rate (dashed lines) and from the maximum heterologous fraction, ${\varphi }^{\text{max}}_\text{H}$ (solid lines). The similarity of the results obtained through these two different methods in the case of the DE3 system suggests that the expression fractions are indeed constant within these intervals, including the beginning of the stationary phase, where the values of ${\varphi}^{\text{max}}_\text{H}$ were identified.

Furthermore, it will be important to compare the Hill parameters ($H,\ k_I,n$) obtained by fitting the *f*_H_ values from both the maximum method and the slope method.

### 3.5 Application on other biosensors (non-T7RNAP)

The DE3 expression system depends on T7RNAP-driven transcription, which is suitable for various applications but somewhat restricted in terms of regulatory possibilities. In contrast, numerous transcription factors and other regulatory elements have been identified in relation to bacterial RNAP. To evaluate the broader applicability of our methods, we examined inducer titration experiments from two additional systems using transcription by *E. coli* RNAP.

**Figure 5.**
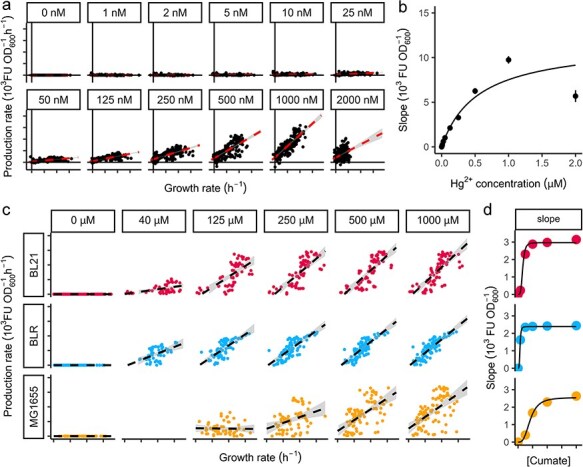
Using the slope method for non-T7RNAP biosensors. (A) Production vs growth plots for the MerR system in response to ionic mercury ranging from 0 to 2000 $\mathrm{nM\ HgBr}_{2}$ in $\mathrm{NEB}^{\circledR}$Stable cells. Dots are experimental data fitted to a linear regression (dashed line). (B) Dose–reponse diagram obtained by using the numerical value of the slopes obtained in the linear regressions. The black line is a Hill function fitted into the dose–response plot. (C) Production vs growth plots for different cumate concentrations stimulating the CymR–cumate system in three different *E. coli* chassis. Dots are experimental data fitted to a linear function (dashed line) using linear regression. Unlike the DE3 system, excluding early points did not improve the linearity of the plots for both CymR–cumate and MerR–mercury systems, implying a faster equilibration of these inducers as discussed in the text. (D) Dose–reponse diagrams of each strain using the numerical value of the slopes obtained in the linear regressions. A Hill function (black line) was fitted in each of the dose–response graphs.

First, a mercury biosensor was constructed based on the MerR transcription factor (complete characterization in [38]). Upon ionic mercury titration, we observed that the reporter protein accumulated continuously, even during the stationary phase; therefore, we were unable to identify ${\varphi }^{\text{max}}_\text{H}$. However, the specific production vs. specific growth rate graphs (Figure 5a) produced linear shapes. A linear trend was detected in the plots and was approximated to a single slope that intersected at zero. A dose–response diagram was plotted using the expression fractions obtained from these slopes and was fitted to a Hill function as shown in Figure 5b. The estimated parameters for this Hill function can be found in Table 1.

In addition, a cumate biosensor was built based on the constitutive expression of CymR repressor as seen in previous reports [39]. In the case of cultures induced with cumate, the reporter protein production decreased earlier than the arrest of growth, that is, before the onset of the stationary phase. Consequently, a ${\varphi }^{\text{max}}_\text{H}$ was readily identified. Also, when observing the specific production vs specific growth rate scatterplots in Figure 5c, we noted that the graphs did not exhibit an initial lapse of nonlinear behaviour, indicating that the intracellular concentration of cumate equilibrates relatively rapidly. Like before, we plotted a dose–response diagram using the slopes of the production vs. growth plots and fitted a Hill function (Figure 5d). The calculated parameters are listed in Table 1.

An additional observation is that the linear lapse in the relation between specific production and specific growth rates showed an intersection different from zero on the specific growth rate axis, thus representing the period when specific production ceased while growth continued (*µ* > 0). Therefore, the regression in the cumate system must include the value of this intersect, thus implying that the equation for the specific production rate *ρ* is


(7) $$ {\rho }_\text{H}(t)=f_\text{H}(t)\cdot [\mu(t) - b]. $$

We anticipate that the intersection ${f}_\text{H}(t) \cdot b$ will be present in other biosensors when the maximum heterologous protein fraction (${\varphi }^{\text{max}}_\text{H}$) occurs at a positive specific growth rate (*µ* > 0). In systems like the CymR–cumate system, where the specific growth rate at the zero specific production rate intersection is significant (comparable to the slope value), it is no longer true that the maximum ${\varphi }^{\text{max}}_\text{H}$ equals ${f}_\text{H}$. Therefore, the regulatory parameters extracted from the dose–response plots will differ when using the ${\varphi }^{\text{max}}_\text{H}$ method compared to the slope method. For illustrative purposes, Figure 2S presents Hill plots generated by both methods. However, only the expression fractions obtained from the slope method should be considered reliable.

## 4 Discussion

This research tackles the challenge of standardizing methods for characterizing regulatory circuits and their modular components across various cellular contexts. The consistent evaluation of these parts is crucial for the long-term goal of designing predictable biological systems based on well-understood individual components. To this aim, we utilize conceptual tools from bacterial physiology to facilitate the characterization of sensing circuits, particularly focusing on transcription factor-based biosensors.

The conceptualization of the cell as a limited proteomic space introduced by Scott *et al.* [26] (Figure 2) and expanded by subsequent kinetic models [27, 33, 57] offers a useful theoretical framework to study physiological phenomena and links it to genetic regulation mechanisms [7]. In this article, by using the time derivative of the formal definition of a proteome fraction, the focus is shifted from protein yield to protein biosynthesis rate. Following the two alternative methods presented here, the experimental results readily produce the expression fraction, which represents a protein-specific production rate. By means of inducer titrations, the experimenters may obtain the expression fraction at various inducer concentrations, which are then summarized in a single set of Hill function parameters.

We first evaluated our approach by characterizing the DE3 system in three distinct strains, considering both the presence and the absence of additional expression of the LacI repressor. Although the slope and maximum methods yield reasonably similar results, it is evident that these parameters are significantly influenced by the cellular context. For instance, the hooked shapes in the rate graphs in Figure 4a illustrate the time interval during which the expression fraction is not constant, presumably because the inducer has not yet reached equilibrium with the transcription factor. It is worth noting that the maximum growth rate may occur during this non-equilibrated time. This lag period is shorter for higher concentrations of the inducer, as one would expect from a diffusion-dependent equilibration. Furthermore, it seems to be strain dependent and is associated with B strains exhibiting a higher apparent affinity for the inducer, as shown by a lower value of *k*_I_ than MG1655(DE3). These variations cannot be attributed to actual differences in the association reaction between the LacI protein and IPTG; therefore, these observations could be attributed to variations in IPTG permeability, considering the regulation of lactose permease (LacY) and the relative contribution of passive diffusion and active transport among these strains. A K-12 strain has been reported to depend on inducible LacY for the internalisation of IPTG [58, 59]. In agreement with this, when LacI overexpression was introduced in MG1655(DE3), *k*_I_ increased three or four times its value, which means that the apparent affinity of the system decreased, probably due to the reduced internalization of IPTG caused by decreased LacY expression. This was not the case for the BLR(DE3) strain, where LacI overexpression did not affect the apparent *k*_I_. Although we were unable to find information on IPTG permability in B strains, they are considered to have higher general permeability than K-12 [60]. This difference in permeability could explain the contrasting responses observed between the two strains. Additionally, the overall effect of additional LacI expression via pACYC was more subtle in BLR(DE3) compared to the K-12 strain, MG1655(DE3), which was severely burdened, resulting in significantly lower growth and fluorescence production. Such careful analysis of the rate plot and Hill parameters in different contexts provides valuable observations specific to the cells where the system is tested. In future studies, we plan to evaluate and deepen into more biosensor cases, including the biosensors for mercury and cumate presented here, to see in what cases the Hill parameters are kept across various cells and in what cases the variations are informative of strain-specific characteristics.

We evaluated the general applicability of the two analytical methods (maximum and slope) by analysing titration experiments from two additional biosensing systems. These systems detect cumate and mercury using CymR and MerR transcription factors, respectively. The maximal fraction method was not suitable for the MerR system because the reporter protein continuously accumulated, even after entering the stationary phase, preventing the detection of a clear maximum point ${\varphi }^{\text{max}}_\text{H}$. For the cumate biosensor, although the maximal fractions were readily identifiable, the presence of an intercept implies that ${\varphi }^{\text{max}}_\text{H}$ does not equal the expression fraction. This discrepancy explains the inconsistencies between the Hill parameters obtained for the CymR system from the maximum and slope methods. Despite these limitations with the maximum method, the slope method remained a reliable approach for both the CymR and MerR systems. The extended linear regions observed in the rates plots indicated the suitability of these systems for analysis using the slope method. Furthermore, the brief non-linear phase suggests a faster equilibration with their specific inducers compared to the slower equilibration observed with IPTG.

In addition to analysing their applicability conditions, we also consider the limitations of each method that should be acknowledged for future uses. The maximum fraction method relies on a single data point, often obtained during the stationary phase. This approach does not provide information on the interval of time at which the expression fraction thus determined is valid. Furthermore, cellular functions are downregulated during the stationary phase, potentially compromising the suitability of the method for assessing the biotechnological applications of a system despite a high apparent heterologous expression level. In the case of the slope method, it employs a linear regression in a time window of constant slope in the data, thereby overcoming this limitation of the maximum method. However, the ranges of linear correlations must be identified for each analysed culture condition, which involves a potential source of error. In this method, the selection of points for the regression analysis might be subjective or insufficient if dealing with a small number of data points. To address this limitation, we propose defining strict data requirements and implementing an algorithm for a more objective determination of the linear range. Other improvements will arise from better protein measurement accuracy. Here, we approximated the reporter proteome fraction, $\varphi_\text{H}$, simply normalizing the fluorescence by $\mathrm{OD}_{600}$. Better measurements can be made by complementary techniques, such as relating the registered fluorescence intensity to specific amounts of reporter protein through a calibration curve and mass spectrometry proteomic approaches. In broad terms, this work presents methods and concepts designed to aid researchers in applying principles from advanced metabolic studies to characterize new regulatory elements and develop novel biosensor circuits. For example, a significant challenge in biocircuitry design is the lack of formal evaluation of orthogonality between heterologous gene expression and native functions [3, 61]. Existing methods often lack standardization and rely on comprehensive and expensive techniques such as RNAseq and ribosome profiling [62–65]. Alternatively, researchers employ specific, hypothesis-driven tests to identify interference between regulatory components [66–69]. In contrast, the framework presented here offers a relatively simple approach to assess the regulations of native functions, composed of expression fractions *f*_C_ (nutrient fixators) and *f*_R_ (biomass producers), are influenced by *f*_H_ (heterologous expression). For example, researchers can manipulate the carbon sources in the culture to observe changes in *f*_C_ [26, 33, 57]. Alternatively, physical or biochemical stress mediated by ppGpp can be used to monitor changes in *f*_R_ [27, 70, 71]. These manipulations activate distinct cellular regulatory mechanisms and, if a novel heterologous circuit affects these mechanisms, a dose–response relationship should emerge, with increased heterologous expression correlating with heightened interference. This approach could reveal interactions between native metabolic regulations or stress responses and the heterologous gene(s) as it has been previously proposed [7]. In this way, the concepts of proteome fractions and expression fractions offer a more nuanced approach to evaluate reporter protein production compared to simple normalization by culture density or growth rate. These methods remain cost-effective and convenient, potentially encouraging wider adoption of a systems-wide approach in the characterization of biomolecular parts for synthetic biology and improving reproducibility across laboratories.

## Supplementary Material

ysaf002_Supp

## Data Availability

Datasets are available on FigShare: https://doi.org/10.6084/m9.figshare.26314723.v1. The sequences of the genetic circuits designed for this study are available on GenBank: IPTG biosensor (GenBank: PQ015608) and the cumate biosensor (GenBank: PQ010742). The sequence of the mercury biosensor is described in [38]. All plasmids used in this study will be shared upon request to the corresponding author.
