# Supplementary material for: Biosensor characterization: formal methods from the perspective of proteome fractions
Source: Synth Biol (Oxf). 2025 Feb 12;10(1):ysaf002. doi: 10.1093/synbio/ysaf002 (PMC11826058; doi:10.1093/synbio/ysaf002)
Supplement: ysaf002_Supp [file ysaf002_supp.zip › suppl_data/Data_Availability_statement.docx]

**Data Availability statement**

| \| \| \| \| \| \| \| \| \| \| **Yes** \| **No** \| **Does the manuscript use or report the following? If so, please provide details in a Data Availability statement below and in the manuscript.** \| \| --- \| --- \| --- \| \| yes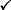 \|  \| 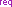 Novel nucleic acid sequences including sequences of new plasmids - Must deposit in EMBL / GenBank / DDBJ. - Must provide sequence names and accession numbers or upload GenBank files in the article online supplement. Plasmid sequences should NOT be embedded in Word or PDF files. \| \|  \| 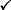 \| 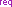 Synthetic nucleic acid oligonucleotides including PCR primers, gRNA, siRNAs or shRNAs - Manuscript should provide exact sequences, exact details of chemical modifications at any position, and source of reagent or precise methods for creation. - Sequences should be provided as multiple sequence FASTA file or comma-delimited text files. - These can be included in the main text or in Supplementary Material. \| \|  \| 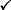 \| 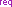 Mass spectrometry proteomics - Must deposit to ProteomeXchange consortium and provide Dataset Identifier and reviewer account details. \| \|  \| 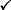 \| 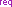 New genome expression or sequencing data (ChIP-seq, RNA-seq…) - Must comply with ENCODE Guidelines, deposit data in GEO, and view data on the UCSC (eukaryotes) or Artemis (prokaryotes) sequence browsers. - Must provide GEO accession numbers and private tokens for Referees and UCSC or Artemis genome browser session links (even if GEO entries are publicly available). \| \|  \| 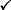 \| 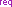 Nextgen sequencing reads including Illumina, PacBio, Oxford Nanopore - Must Submit sequencing reads to SRA, ArrayExpress or GEO. - Must provide accession numbers and private tokens (GEO) or login details (ArrayExpress). \| \|  \| 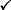 \| 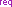 Microscopy images including series of time-lapse microscopy images should be deposited with The Cell Image Library. - Must provide accession number. \| \|  \| 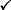 \| 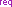 Novel nucleic acids structures - Must deposit to NDB (via PDB if possible) and provide accession details. \| \|  \| 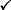 \| 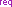 Novel protein sequences including sequences of engineered proteins - Must submit to UniProt using the interactive tool SPIN and provide sequence names and accession number. \| \|  \| 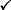 \| 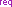 Quantitative PCR - Must comply with the MIQE Guidelines. - Details should be supplied in Materials and Methods section of manuscript. \| \|  \| 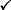 \| 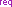 Flow Cytometry experiments - Must deposit in FlowRepository. - Must provide Repository ID and secret code for Referees. \| \|  \| 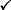 \| 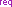 Source code of open source software - Should be deposited in a publicly available repository (GitHub, SourceForge, BitBucket). - Source code should include an OSI-approved license. - Non-open source software including commercial software should be accessible anonymously and at no cost to the reviewers. \| \| 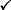 \|  \| 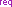 Gel images, micrographs, graphs, and tables may be included in the article online supplement. Optionally, they may be deposited in a general-purpose repository such as Zenodo, Figshare, or Dryad. \| \| \| --- \| --- \| --- \| --- \| --- \| --- \| --- \| --- \| --- \| --- \| --- \| --- \| --- \| --- \| --- \| --- \| --- \| --- \| --- \| --- \| --- \| --- \| --- \| --- \| --- \| --- \| --- \| --- \| --- \| --- \| --- \| --- \| --- \| --- \| --- \| --- \| --- \| --- \| --- \| --- \| \| \| --- \| --- \| --- \| --- \| --- \| --- \| --- \| --- \| --- \| --- \| --- \| --- \| --- \| --- \| --- \| --- \| --- \| --- \| --- \| --- \| --- \| --- \| --- \| --- \| --- \| --- \| --- \| --- \| --- \| --- \| --- \| --- \| --- \| --- \| --- \| --- \| --- \| --- \| --- \| --- \| --- \| \| \| --- \| --- \| --- \| --- \| --- \| --- \| --- \| --- \| --- \| --- \| --- \| --- \| --- \| --- \| --- \| --- \| --- \| --- \| --- \| --- \| --- \| --- \| --- \| --- \| --- \| --- \| --- \| --- \| --- \| --- \| --- \| --- \| --- \| --- \| --- \| --- \| --- \| --- \| --- \| --- \| --- \| --- \| \| \| --- \| --- \| --- \| --- \| --- \| --- \| --- \| --- \| --- \| --- \| --- \| --- \| --- \| --- \| --- \| --- \| --- \| --- \| --- \| --- \| --- \| --- \| --- \| --- \| --- \| --- \| --- \| --- \| --- \| --- \| --- \| --- \| --- \| --- \| --- \| --- \| --- \| --- \| --- \| --- \| --- \| --- \| --- \| \| \| --- \| --- \| --- \| --- \| --- \| --- \| --- \| --- \| --- \| --- \| --- \| --- \| --- \| --- \| --- \| --- \| --- \| --- \| --- \| --- \| --- \| --- \| --- \| --- \| --- \| --- \| --- \| --- \| --- \| --- \| --- \| --- \| --- \| --- \| --- \| --- \| --- \| --- \| --- \| --- \| --- \| --- \| --- \| --- \| \| \| --- \| --- \| --- \| --- \| --- \| --- \| --- \| --- \| --- \| --- \| --- \| --- \| --- \| --- \| --- \| --- \| --- \| --- \| --- \| --- \| --- \| --- \| --- \| --- \| --- \| --- \| --- \| --- \| --- \| --- \| --- \| --- \| --- \| --- \| --- \| --- \| --- \| --- \| --- \| --- \| --- \| --- \| --- \| --- \| --- \| \| \| --- \| --- \| --- \| --- \| --- \| --- \| --- \| --- \| --- \| --- \| --- \| --- \| --- \| --- \| --- \| --- \| --- \| --- \| --- \| --- \| --- \| --- \| --- \| --- \| --- \| --- \| --- \| --- \| --- \| --- \| --- \| --- \| --- \| --- \| --- \| --- \| --- \| --- \| --- \| --- \| --- \| --- \| --- \| --- \| --- \| --- \| \| \| --- \| --- \| --- \| --- \| --- \| --- \| --- \| --- \| --- \| --- \| --- \| --- \| --- \| --- \| --- \| --- \| --- \| --- \| --- \| --- \| --- \| --- \| --- \| --- \| --- \| --- \| --- \| --- \| --- \| --- \| --- \| --- \| --- \| --- \| --- \| --- \| --- \| --- \| --- \| --- \| --- \| --- \| --- \| --- \| --- \| --- \| --- \| \| \| --- \| --- \| --- \| --- \| --- \| --- \| --- \| --- \| --- \| --- \| --- \| --- \| --- \| --- \| --- \| --- \| --- \| --- \| --- \| --- \| --- \| --- \| --- \| --- \| --- \| --- \| --- \| --- \| --- \| --- \| --- \| --- \| --- \| --- \| --- \| --- \| --- \| --- \| --- \| --- \| --- \| --- \| --- \| --- \| --- \| --- \| --- \| --- \| |
| --- | --- | --- | --- | --- | --- | --- | --- | --- | --- | --- | --- | --- | --- | --- | --- | --- | --- | --- | --- | --- | --- | --- | --- | --- | --- | --- | --- | --- | --- | --- | --- | --- | --- | --- | --- | --- | --- | --- | --- | --- | --- | --- | --- | --- | --- | --- | --- | --- |
